# Doctorate Address Delivered at the Commencement of the Woman’s Medical College

**Published:** 1884-06

**Authors:** Wm. H. Byford

**Affiliations:** President


CHICAGO MEDICAL

JOURNAL & EXAMINER.

VOL. XLVIII.—JUNE, 1884.—NO. 6.

### Original Communications.

### ARTICLE I.

DOCTORATE ADDRESS Delivered at the Commencement of the Woman's Medical College, by WM. H. BYFORD, A.M., M.D., President, on April 22, 1884.

*Ladies and Gentlemen :*—The Faculty of the Woman's Medical College have kindly requested me to represent them before this audience.

I feel flattered by this request, and regret that my ability for the discharge of such a duty is not as great as I could desire. I accept it, however, because I have something I want to say to the non-professional portion of this assembly, on the subject of woman's fitness for the practice of medicine, and the propriety of her doing so.

I admit that there is not much need of argument in favor of a demonstrated fact; but I am also aware that there yet remains much prejudice in the minds of many honest people against medical women.

The propriety and necessity of women entering the profession of medicine, and indeed, other avocations heretofore occupied by men, are but the results of changes in our social fabric which have been going on for a long time. The position of woman in her relations to society, is not what it was a half century ago, and can never be restored to her. Many of the agencies that have brought this about, are still at work, and their future effects will be irresistible. Many of us remember the time when every woman could look forward to the possession of a home, and to a time when she could exercise those functions for which by nature she was intended, and in which she could enjoy the preëminent blessings of domestic life. Her training led up to those grandest of all duties, the moulding and education of her generation.

In her was centered, to a greater extent than any other being, the happiness and prosperity of mankind. Unfortunately, with the march of events, the conditions under which such a life was practicable, have been swept from society, and hundreds of thousands of women cannot aspire to domestic duties.

It is a curious fact, that much of this change has been brought about by the great advance made in modern civilization. Labor-saving machinery, the result of science applied to the every day affairs of life, does the spinning, weaving, sewing, knitting, washing, and a great many other kinds of work formerly done by women.

This, of course, relieves women of a great deal of drudgery, and in a certain sense improves the condition of those who can live without laboring for their own support, but it leaves others unable to earn a living in occupations recognized as belonging to them.

Then again, many of the duties devolving upon womankind are allotted to the opposite sex. The male cook, house servant, and laundryman, are too common to require mention, and scarcely less numerous are male milliners, and mantua-makers. It would be tedious to recite all the usurpations of the scientific workers of the sterner sex. I say, scientific workers, because even cooking and washing, as well as many other domestic processes, are done in accordance with chemical and mathematical principles.

I do not wish to be understood as complaining of the unquestionably great improvements of the times. They are no doubt, designed to, and will work out beneficent results for the race, but until society accommodates itself to the changes, we must expect some of its members to suffer on account of their unfavorable position in social life. We should not, perhaps, desire a restoration to women of all their former occupations, but should seek for them, in the new and better state of things, more profitable, if not more congenial avocations.

Another question relevant to the subject of the changes now going on in society, is matrimony. The order of things in time past, made the obligation to marry imperative, and the stigma "old maid" was a galling taunt to every sensitive woman. Even now, the most desirable thing for every good woman is to marry, and I am sure I will not be wide of truth in stating that almost, if not quite all women, look upon the undivided devotion of one good man as the main element of an earthly paradise. And they are right. But, in the very nature of things, many women cannot marry. A number of reasons will present themselves in support of this statement. There is a great numerical disparity of the sexes. The marriageable women of this country exceed the number of men who *might* enter the bonds of matrimony. Then there is a growing disposition among men not to marry. Why should this be? There are many good causes, when we look upon the subject from the standpoint of the social changes going on. Every American whose love of liberty depends very greatly upon his ability to stand square before society with every other American, has not the means to support himself and a wife in first-class style. His desire is, very naturally, that his wife should be as accomplished and beautiful as any other woman living. He must therefore support her and an establishment in accordance with his views, or he will not marry. Born and educated in society as it now is, who can blame him? The majority of women look for this kind of a man. Society, you know, demands this kind of respectability, or seclusion. While he lives in a hotel, or club-house, dresses well, and behaves himself with propriety, society receives him with open arms. If he marries, and cannot keep up appearances, it shuns him. Sometimes good, honest, affectionate men marry and take their wives to hotels or boarding houses, and spend all their earnings to keep up appearances; but too frequently, this artificial and strained mode of life leads to conjugal infelicity, instead of domestic happiness.

Should one of these men have the courage to marry and live in the humble manner warranted by his income, his gilded surroundings often lead to discontent.

And again, how the male vagabonds in this land are multiplying, men whom no good woman can marry and maintain her self-respect! These things are a part of the great changes now transpiring in the social world.

These, and like circumstances, are so numerous as to compel women to look out for independent means of support, and they are showing themselves equal to the task. The doors of domestic life are closed against so many of them, that they would be reckless if they did not seek some occupation that would enable them to live honestly, respectably, and independently.

It is not my purpose or desire to advocate the political rights of woman, nor to express my opinion upon this subject, which all must see is becoming an important problem of the age, but I am here to say that it is the sacred duty of us all to promote every enterprise that gives her an equal chance for an honest independence. She deserves such equality, and society will not be vindicated in her almost revolutionary progress until that end is attained.

The medical education of women is one of the means calculated to promote that very desirable object. There are still lingering among us, some persons who object to female practitioners of medicine. They urge objections which are based upon unfounded sentiment, instead of good sense and logic. The foundation for valid objections has long since ceased to exist. Such objectors believe that this and kindred movements will have the effect of undermining that good old institution we call "home."

No man has more veneration for the sacred relations existing among members of a well-regulated home, than I have. No man would go farther to foster that more than human institution, with all its precious belongings, as it was, and now is, than I would. No man reveres the holy relationship of mother, the loving and warm-hearted wife, or the confiding, worshipping dependence of a daughter more than I do. But all this laudable sentimentality about home should not blind us to the fact that there are now, and in the future must remain thousands upon thousands of women to whom none of these relations can come.

There are many men who affect to believe that the study of medicine has a degrading influence upon the pure minds and hearts of the sex, and that the practice of that almost divine art places her outside the pale of propriety. Useful knowledge is always elevating to its possessor, and whatever her future destiny may become, woman is purer and wiser, because of a knowledge of the physical and mental attributes of human beings.

Regarding the honored positions of mother and wife, as I do, as the most elevated any good woman can occupy, I consider that only a very little lower is that of the woman to whom are entrusted the lives of the mother and her children.

But is woman capable of discharging the mighty obligations incumbent upon practitioners of medicine? Has she the physical strength, nerve, and endurance, and has she the brains? These are practical and not sentimental questions, questions that must be answered by an array of facts instead of imaginative theories.

In traveling through Belgium and Austria, I think I saw the first part of this interrogatory answered, in a very emphatic, if not gallant manner, where woman is compelled to try her strength with a donkey, by being hitched by his side to a heavy cart.

On the way from Brussels to Waterloo, on the military highway built by the despotic Emperor of France for the subjugation and maintenance of his authority over the people who have so degraded womankind, may be seen every few rods such a team as this, tugging and trudging along before a cart filled with manure to enrich the soil, upon which the two working together may raise vegetables, to be brought to market in the same manner.

In the Austrian capital I have seen women carrying large hods of brick and mortar on rickety ladders to the top of the tallest structures. They did this while a representative of the lords of creation stood around to direct aright the muscular energies of these stalwart females.

It is a well-known fact, that in many of the old countries in Europe the women do the most laborious portion of farm duties. These exhibitions of physical power and endurance cannot fail to impress the observer with a better opinion of the capabilities of the sex to bear the toil and fatigues of the profession that tries men's muscle and nerve.

But a fact more to the point may be found in the habits of the pioneers of this country, and it is pertinent, because it is an experiment elucidating the endurance of woman in the very duties to which her professional education calls her. When this and the immediately neighboring States were the western frontier, sparsely settled, covered with marshes, prairies, and forests, swept by the coldest of winds in winter, and parched by the hot sun of summer, there was a class of women called midwives, whose avocation and responsibilities were of the most trying character. They traveled by day and night, through the wet and cold, in the unceasing demands of their calling, and maintained a physical condition equal to every occasion that might present itself. I know that many of them did more of this irregular and wearing business than usually falls to the lot of the popular practitioner of the city or country. Often they themselves were mothers of large families, and were full of domestic obligations.

Even now, in this city, I could point to women who are enduring the ordeal of a full practice in this specialty, with all its trials, better than the majority of men. Those whose experience has placed them in a position to know, will assure you that there is no branch in the medical profession so exacting in toil, self-denial, and responsibility as this, and yet it is the one which has always largely devolved upon women. Is it not absurd, then, in view of these facts, to say that woman has not the physical ability to practice medicine? That objection is a subterfuge under which to cover the prejudices of the objector, or if honestly entertained, it is for want of definite information upon the subject, and consequently, proves itself to be mere ignorant prejudice.

The question is not whether women, as a class, are as strong as men, but whether they possess the strength necessary to become efficient practitioners. In view of the facts of the past and the present, I can have no doubt they are.

But, has she the nerve and courage equal to the appalling emergencies which sometimes unexpectedly present themselves? Has she the daring and dash of a bold surgeon? is she capable of doing the right thing at the right time, with that calm self-possession so necessary to carry her through great surgical operations? Let us see. What is surgical courage? Is it a natural gift possessed by a few favored ones? I am sure it is not. It is an acquisition to which the best of us attain through an elaborate process of training. It is one of the results of a thorough medical education. It does not come to the strongest of us except in that way. Is this not true in every vocation requiring courage? Green recruits are not the reliable support of an army. The veterans only are an unflinching resistance to the advance of a powerful foe. Let the soldier tell you whether he was not strongly inclined to conceal himself or fly from the enemy in his first battle. Even the veteran hero of Waterloo, once said that he never entered a battle without trepidation, and that he never exposed himself to danger except from a sense of duty, and that natural bravery did not enter into his composition. Yet he never was suspected of cowardice, nor was he a coward, but he had acquired the courage necessary for a great general, and capacity of becoming the conqueror of Napoleon, through military training.

Surgical courage, technically speaking, is not a congenital trait of character. No one knows this better than the veteran surgeon. Now, in reference to courage in our profession, every surgeon remembers the harrowing episodes of his early career; with what misgiving, apprehension, and trepidation, he approached the lying-in chamber, or grave surgical operation, and how exhausted after he had passed through any considerable difficulty in either situation. It takes a long time to completely overcome the solicitude of the surgeon who is preparing to do a great surgical operation, which may decide the fate of a fellow being. And in this respect, all are alike, all are more or less discomposed when first entering the practical part of professional life.

In the very nature of a medical education, there is discipline that gradually leads the new recruits up to the degree of courage necessary for action. The dry skeleton commences the drill. Its naked squalor, the ugly grin with which it greets you, not only its teeth, but every bone in its ghastly countenance express more impressively than actual words, the presence of death. The very sight of it causes the uninitiated to recoil in breathless horror. The student soon learns to scan every part of it with the intensest interest, and familiarly, indeed almost lovingly to nurse it into the late hours of night, and when all its mysteries are mastered, it is laid aside for the cultivation of subjects a grade higher in ghastliness. These are found in the dissecting room. The feelings of every individual when first entering that place are simply indescribable. Nothing but the knowledge that it is the ante room to physiology and pathology,—the processes of life and death—could reconcile the student to return to the dreadful scenes there displayed with such ghastly reality. Yet that place becomes so attractive that one forgets all its horrors, in the sublime glimpses it affords of the workings of the Great Author of our being. And after a time, the timid woman will fondly gaze into this mirror of humanity, and regret when obliged to leave it for still further advances in the study of her glorious art. Indeed, so captivating and so necessary is the study of anatomy, that she will often recur to it, and stand absorbed in its revelations with a pleasure incomprehensible to those who have not passed through such experiences. This is an exhibition of professional courage that may well be regarded as a great step in her march towards victory over her natural timidity.

The autopsy now is an inspiration to her—it is an exhibition of the traces drawn by the hand of her grim enemy upon his victim, and teaches her the methods of his approaches and attacks upon the citadel of life. She is thus armed with the knowledge which will enable her to parry his thrusts, and baffle his deadly onslaughts upon our nature.

In the hospital she becomes familiar with the anatomy of the living, every conceivable form of bloody mutilation, and the expression of pain, suffering, and death. She participates in the ministrations of the hospital staff, aiding the surgeon, and sometimes performing amputations, the removal of tumors, and other operations, until she knows all the methods, and has acquired—what people call "hard-heartedness"—but which is surgical courage enough to look with calmness upon scenes which fill with terror her uninitiated sister. Amid these scenes in which she is an actor, she acquires the strength of nerve, and the habit of composure, that enables her to do her duty in every trial with promptitude and precision.

That women are equal to all the emergencies of the most important surgical and obstetrical operations, we have ample proof in this city and elswhere. There are scores of women surgeons in this country who have repeatedly done the most daring and difficult surgical operations, and the number of individuals educated up to that point grows larger. I have many times witnessed the exhibition of this surgical capacity and coolness on the part of women in this city. I could point out to this audience women practitioners who do not hesitate to undertake, and who accomplish surgical achievements of which any man might be proud. I will venture to assert, what is generally known to the profession in Chicago, that one of our alumnæ left as brilliant a surgical record in Cook County Hospital as any young man who preceded her. And the young male interne who shall excel her in boldness and dexterity in that branch of the healing art, will some day win for himself position and renown.

Medical women have successfully embarked in the literature of the profession. Several of them have written books that are regarded very justly as eminently respectable authority on the subjects upon which they treat, and every month well-written papers from them adorn the pages of the medical journals of this country.

In the medical colleges devoted to the education of the sex, women are chosen for, and very acceptably discharge the duties of teachers and professors, and with a continuation of the progress they are now making, it will require but a few years to render these institutions as they ought to be — entirely independent of medical men for their corps of teachers.

As practitioners, the people of this city can judge for themselves. How many women are now enjoying the confidence of the people, and are engaged in lucrative professional practice, I will not undertake to say, but we all know of a score or more, and they are becoming more numerous every year. The prejudices which have been transmitted from one generation to another for a long time, are rapidly giving way under the new state of things. The intelligent observer who has been cognizant of the changes which have been going forward for the last twenty years, can easily appreciate the causes which are dissipating this firmly-rooted prejudice against women.

Before women were admitted to the medical colleges, and permitted to enjoy the privileges these institutions afford them, the women who practiced medicine were ignorant, and in most instances, unscrupulous pretenders, and deserved the condemnation of the people and the profession. Those who were honest, and had the good of their patients at heart, were debarred from the opportunities of obtaining a thorough medical education. Their chances for knowledge were confined to the reading of a few antiquated books, and the occasional opportunities for observations they were so fortunate as to be blessed with. In addition to their entire absence of thorough teaching, many of them were of a very low type of womankind. To do justice even to this class of pretenders, however, it ought to be said that they had their professional prototypes in the opposite sex, and that scores of men practiced medicine who were equally ignorant and unscrupulous, for whom there is no valid excuse, as they had free access to the medical colleges at all times. Now, thanks to medical legislation, both of these classes of charlatans are driven from our profession, and both sexes enjoy equality in the opportunities to obtain a medical education. Women not only have the opportunities to study and become scientific practitioners, but the standard of the schools to which they are admitted is as high, and the requirements as rigid as in colleges graduating men. Their sex affords them no immunity from the same exacting examinations for the degree of Doctor of Medicine, and they do not expect or desire that it should. We find them bright, intelligent, and earnest students, and they pass examinations that would entitle them to the honors of any medical college in the country. This places them in the category of scientific physicians, and upon the same plane of standing with medical men.

The intelligent members of society are becoming aware of these changes in the circumstances under which women are educated, and consequently, the qualities of women practitioners. The result is, they appreciate them more, and are employing them with more freedom, and giving them that implicit confidence to which every competent and faithful physician is entitled.

Now, having witnessed what they have done for themselves in a little over two decades of time, I think we can justly say that they have achieved an honorable beginning, and can reasonably predict for them a permanent and enviable position in the profession for all time to come.

### TO THE CLASS OF GRADUATES.

How natural it is for us when we are graduated to look upon the knowledge imparted to us as our own, and think of it as the result of our own hard study. This would be a very great mistake for you to make. You have been studying three years, and have been successful in fixing in your minds a small modicum of professional knowledge. You have not made any of the discoveries of facts upon which that knowledge is based. Millions of the best men of all time have been for many centuries constantly engaged in analyzing, demonstrating, and arranging the facts and knowledge which have been imparted to you in the last three years. Imagine yourselves searching for the information necessary to fit you for the practice of medicine without the light thus afforded, and you will realize how much you owe to your predecessors, and how little credit is due you in the work of accumulation. The laborers of the past ages, who have thus faithfully and ardently worked to acquire knowledge, and those of the present time who are still pushing forward, surveying every field and path of science in which facts relevant to their own may be found, constitute the profession of medicine.

To this body of worthy workers you owe everything you have, all you are, and all you can become professionally.

I say this much to you now, to show you how devoid of reality is that sense of independence upon which the neophyte is apt to base his calculations for the future. While you go with this strong multitude who have through us honored you with a certificate of membership, you will be borne with it, and participate in its triumph. When you turn your back upon it, you will be left in the dark. When you turn your hands against it, you will be crushed in the struggle. Motives of self-interest, as well as the more noble sentiment of gratitude, prompt you to be good and faithful members of that ancient and honored body. Somehow, more is expected of women than of men. While your critics accuse you of being the weaker sex, they are ungenerous enough to exact from you more strength of character than they do from men. They will tolerate no short-comings in you. This, quite as much as the fact that female physicians are still on probation, will put you on your good behavior. Knowing you as well as I do, I welcome the result with the full assurance of triumph. But professional strength is just as necessary for you as professional honor. That comes from much study. The old threadbare saying, "Once a student, always a student," expresses the only condition upon which you can hope to become useful and successful practitioners.

Those richly endowed old friends of yours, to which you have devoted so much attention, that marks and annotations may be found on every page, still possess treasures from which you can draw more knowledge than you have already extracted. At the bedside they will assist you to differentiate the intricacies of disease, to clear up the dark places in pathology, and to treat successfully all curable maladies. If you do not consult them, but trust to the uncertain memories of your student days, their valuable contents will fade from your minds. While the books that have served you thus far are still valuable to you as physicians, you will be obliged to add new ones to your library, and to these some one or two of the best medical journals, and all must be read and assimilated. These new books and journals, if properly studied, will keep you on the tide of progress, and acquaint you with what discoveries in all branches of the healing art are being made in any part of the world. Keep constantly in mind that knowledge will bring you reputation, that it will secure to you a competency for the present, a fund for the days when you are not able to work. It will give you a welcome to all grades of social life, but what is better than all these, it will be the means in your hands of doing good. Doing good is the final object of the profession. Without that object it could not, and ought not to exist, and I need not repeat to you what you have already heard, and will again hear, that the good done by our profession cannot be measured by any finite idea of quantity or quality, but must be left to the award of an infinite hereafter.

To this profession you are under obligations to add something to the store of knowledge which is intended for your successors. As the child can never repay its parents for the love and care so lavishly bestowed, except to give in kind these bountiful blessings to his own offspring, so you cannot absolve your obligations to the profession, except by leaving behind you some new facts or processes of cure. This, too, is the only way to attain to professional immortality. One single imperishable fact will carry the impression you may make upon the world on down the stream of time, until science expires in the coming future dark ages of the world.

There is another view of your obligations to the profession. You know it is but a short time since your claims have been presented for recognition, and for some time difficulties await you at every turn. Now, however, a general welcome is extended to you. That recognition is both public and private. You are admitted to the State, district, county, and city medical societies, and are entitled to all the benefits, both scientific and social, of all these associations. I am rejoiced to tell you also, in this connection, that one of the members of the Faculty of the Woman's Medical College of Chicago can claim the distinguished honor of being the first woman admitted to that great institution, the "American Medical Association." In private practice, you will in every way receive the respect and deference of all medical practitioners. What is better even than all this favorable state of things, is that some of you are the honored wives of medical men, and some more of you are betrothed to medical men. There is probably nothing that in so distinguished a manner carries with it the idea of equality, as the matrimonial alliance. Now that you have passed the curriculum, and are entitled to the open secrets of the college, I am willing to say to you that it is, for the Faculty, a very pleasant thing to educate and prepare one of a couple who have plighted their vows of love to each other, while the man of her choice is attending some other of the excellent medical institutions of the city. We always expect to have the best graduate.

A valedictory address is usually a farewell address, in which we are supposed to indulge in lachrymose demonstrations of sorrow at parting. My address, however, is one of welcome, and I rejoice far more than I lament that you are going home to mingle with friends, who are much dearer to you than we are.

Hitherto I have lectured to you as students, who were dependent upon the Faculty of this college for instruction and advice. I now address you as doctors, as peers in titulary dignity, and in the name of the one hundred thousand members of the profession in the United States, I extend to you the right hand of fellowship.

I do this gladly, knowing you, as I do, to be worthy of the honorable association. The Woman's Medical College of Chicago sends you out among those of the profession to whom you are strangers, as its representatives. We do this gladly, knowing you will worthily represent us. We know that in each of you are united the modesty and purity of honored womanhood, to the varied and lofty erudition of Doctors in Medicine. We know that you possess the qualities that will make you ornaments to society, and skilled members of our fraternity. Again I bid you all a most cordial welcome.

